# NLRP3 Inflammasome-Mediated Neuroinflammation and Related Mitochondrial Impairment in Parkinson’s Disease

**DOI:** 10.1007/s12264-023-01023-y

**Published:** 2023-02-09

**Authors:** Qiu-Qin Han, Weidong Le

**Affiliations:** 1 Shanghai University of Medicine and Health Sciences Affiliated Zhoupu Hospital, Shanghai, 201318 China; 2 Center for Clinical and Translational Medicine, Shanghai University of Medicine and Health Sciences, Shanghai, 201318 China

**Keywords:** Parkinson’s disease, NLRP3 inflammasome, Neuroinflammation, Mitochondrial impairment, Microglia

## Abstract

Parkinson’s disease (PD) is a common neurodegenerative disorder caused by the loss of dopamine neurons in the substantia nigra and the formation of Lewy bodies, which are mainly composed of alpha-synuclein fibrils. Alpha-synuclein plays a vital role in the neuroinflammation mediated by the nucleotide-binding oligomerization domain-, leucine-rich repeat-, and pyrin domain-containing protein 3 (NLRP3) inflammasome in PD. A better understanding of the NLRP3 inflammasome-mediated neuroinflammation and the related mitochondrial impairment during PD progression may facilitate the development of promising therapies for PD. This review focuses on the molecular mechanisms underlying NLRP3 inflammasome activation, comprising priming and protein complex assembly, as well as the role of mitochondrial impairment and its subsequent inflammatory effects on the progression of neurodegeneration in PD. In addition, the therapeutic strategies targeting the NLRP3 inflammasome for PD treatment are discussed, including the inhibitors of NLRP3 inflammatory pathways, mitochondria-focused treatments, microRNAs, and other therapeutic compounds.

## Introduction

Parkinson’s disease (PD) is the most common age-related disorder worldwide after Alzheimer’s disease [1]. PD is characterized by the rapid growth of neurodegenerative conditions [2]. Patients with PD show both motor symptoms, such as resting tremor, stiffness, bradykinesia, and muscular rigidity, and non-motor symptoms, such as olfactory impairment, depression, anxiety, fatigue, orthostatic hypotension, gastrointestinal dysfunction, and circadian rhythm dysregulation [3–6]. The pathological hallmarks of PD are the selective loss of midbrain dopamine (DA) neurons and the accumulation of Lewy bodies formed by alpha-synuclein protein in different brain compartments [7–9]. Various treatments such as DA replacement are only partially or transiently effective [3]. As these therapies cannot restore DA neurons to curb the progression of PD, it is critical to find new targets and effective therapies for PD.

Neuroinflammation is one of the most critical factors that contribute to the onset and progression of PD [10]. Both pathological and genetic studies indicate the involvement of neuroinflammation in PD. Postmortem reports and experimental studies have also revealed the roles of both innate and adaptive immunity in the degenerative process [11]. Inflammasomes are important elements associated with neuroinflammation and neurodegenerative disorders [12]. Inflammasomes are multiprotein complexes found in neural cells, microglia, and astrocytes that respond to danger-associated molecular patterns (DAMPs) and pathogen-associated molecular patterns (PAMPs); they induce the release of pro-inflammatory cytokines into the extracellular space. Various inflammasomes play essential roles in neurodegenerative diseases, primarily the nucleotide-binding oligomerization domain (NOD)-, leucine-rich repeat (LRR)-, and pyrin domain (PYD)-containing protein 3 (NLRP3) inflammasome [12]. As the primary immunological cell of the brain, microglia are a major cellular mediator during neuroinflammatory processes, and microglial activation is vital in the neurodegenerative process [13–15]. Activation of the microglial NLRP3 inflammasome and the release of pro-inflammatory cytokines from microglia contribute to PD progression. Impaired DA neurons directly lead to microglial activation, first producing large amounts of pro-inflammatory cytokines and then contributing to the apoptosis of DA neurons. Thus, microglial activation-induced neuroinflammation aggravates the pathological process of ongoing neurodegeneration in PD [8].

In addition, PD progression is associated with mitochondrial impairment, such as disruption of mitochondrial fusion and mitophagy [16]. On the other hand, the impairment of mitochondrial function augments NLRP3 inflammasome activation, triggering systemic or local inflammation [17, 18]. It has been documented that NLRP3 inflammasome-mediated neuroinflammation actively participates in PD progression [19, 20]. However, the relationship between mitochondrial dysfunction and the NLRP3 inflammasome is not well understood. Thus, achieving a better understanding of the interaction between mitochondrial impairment and NLRP3 inflammasome-mediated neuroinflammation in PD progression may facilitate the development of novel therapies for PD. In addition, the therapeutic strategies targeting the NLRP3 inflammasome for PD treatments are discussed in this review, including the application of NLRP3 inflammatory pathway inhibitors, mitochondria-focused treatments, microRNAs, and other therapeutic compounds.

## The NLRP3 Inflammasome

### Formation of the NLRP3 Inflammasome

The components of inflammasomes consist of the apoptosis-associated speck-like protein containing a caspase recruitment domain (ASC) protein, pattern recognition receptors (PRRs), and procaspase-1. There are three types of intracellular PRR: nucleotide-binding domain and leucine-rich repeat-containing receptors (NLRs), absent in melanoma (AIM)-like receptors (ALRs), and the tripartite motif-containing (TRIM) protein pyrin/TRIM20. They are also known to be immunological sensors that can detect DAMPs, PAMPs, and double-stranded DNA in the cytosol [21]. ASC connects the caspase recruitment domain (CARD) of procaspase-1 to the PYD of NLR, ALR, or pyrin [22]. The NLR family includes NLRP1, NLRP2, NLRP3, NLRP6, NLRP7, NLRP12, and NLR family CARD domain-containing 4 (NLRC4) [23]. NLRP3 is activated by PAMPs or DAMPs to form a multimeric protein complex called the NLRP3 inflammasome, a crucial component of the innate immune system.

### Activation of the NLRP3 Inflammasome

Two signaling pathways contribute to NLRP3 inflammasome activation, comprising the non-canonical and canonical pathways [24]. The non-canonical pathway mainly depends on caspase-11 and is mediated by caspase-4, caspase-5, and caspase-8; the canonical pathway predominantly depends on caspase-1. Non-canonical signaling pathways are activated explicitly by Gram-negative bacteria or cytosolic lipopolysaccharides (LPS), inducing the cleavage of gasdermin D (GSDMD) and promoting the activation of the NLRP3 inflammasome and the production of interleukin-1beta (IL-1β) and IL-18, leading to pyroptosis [25, 26].

Generally, activation of the NLRP3 inflammasome in the canonical signaling pathway noted above requires two steps [27]: priming (Step 1) and protein complex assembly (Step 2). Step 1 is first triggered by PRR signals, such as tumor necrosis factor receptor (TNFR) or Toll-like receptor (TLR) activation, and then inducing activation of the nuclear factor-kappa B (NF-kB)-dependent pathway to upregulate the transcription and expression of NLRP3, pro-IL-1β, and pro-IL-18 [28]. Numerous studies have suggested that Step 1 is very complicated, involves transcriptional and post-translational mechanisms, and requires many protein-binding partners. Step 2 is induced by a variety of PAMPs and DAMPs, including extracellular ATP and pore-forming toxins, as well as cellular events, including mitochondrial impairment and the production of reactive oxygen species (ROS), ion flux (K^+^/Cl^−^ efflux and Ca^2+^ influx), and cathepsin B from lysosomal damage [29–31]. For example, there is evidence that K^+^ efflux, known as a reduction of the intracellular K^+^ concentration, is one of the critical cellular events that activate NLRP3 [32]. The activation of NLRP3 can be prevented by maintaining a high extracellular K^+^ state or by inhibiting K^+^ efflux [32]. Many events can induce K^+^ efflux [33]. For instance, after binding extracellular ATP, the ligand-gated ion channel P2X7R opens, allowing K^+^ to pass through. In addition, bacterial toxins, such as valinomycin, nigericin, and gramicidin, form membrane pores in the cell membrane to stimulate K^+^ efflux. In response to K^+^ efflux, NIMA-related kinase 7 (NEK7), a downstream modulator, acts as an important mediator of NLRP3 inflammasome activation through the catalytic domain of NEK7 and the LRR domain of NLRP3 [34–37].

Finally, the inflammasome complex results in the activation of caspase-1, consequently prompting the cleavage of the pro-inflammatory cytokines IL-1β and IL-18 into mature forms and turning the pyroptotic substrate GSDMD into the N-terminus of GSDMD (N-GSDMD) and the C-terminus of GSDMD (C-GSDMD) [38, 39]. The oligomerization of N-GSDMD forms pores in the plasma membrane following cleavage of GSDMD, then mediates the programmed cell death known as pyroptosis, and finally results in the secretion of mature IL-1β and IL-18. It should be noted that, although pyroptosis [40] is similar to apoptosis [41] and necroptosis [42] it, is a type of programmed cell death, and they all differ in morphology [43, 44] and mechanism [45–47]. However, during the apoptosis process, if apoptotic cells cannot be cleared by macrophages, the process changes to caspase-3-mediated pyroptosis [48–50]. Activation of the NLRP3 inflammasome is involved in the pathogenesis of many neurodegenerative disorders, such as PD [51], Huntington’s disease [52], Alzheimer’s disease [53], amyotrophic lateral sclerosis [54], multiple sclerosis [54], and prion diseases [52]. This review focuses on the molecular mechanisms underlying the activation of the NLRP3 inflammasome and its subsequent inflammatory effects on the progression of neurodegeneration in PD.

## NLRP3 Inflammasome-mediated Neuroinflammation in the Pathogenesis of PD

**Fig. 1:**
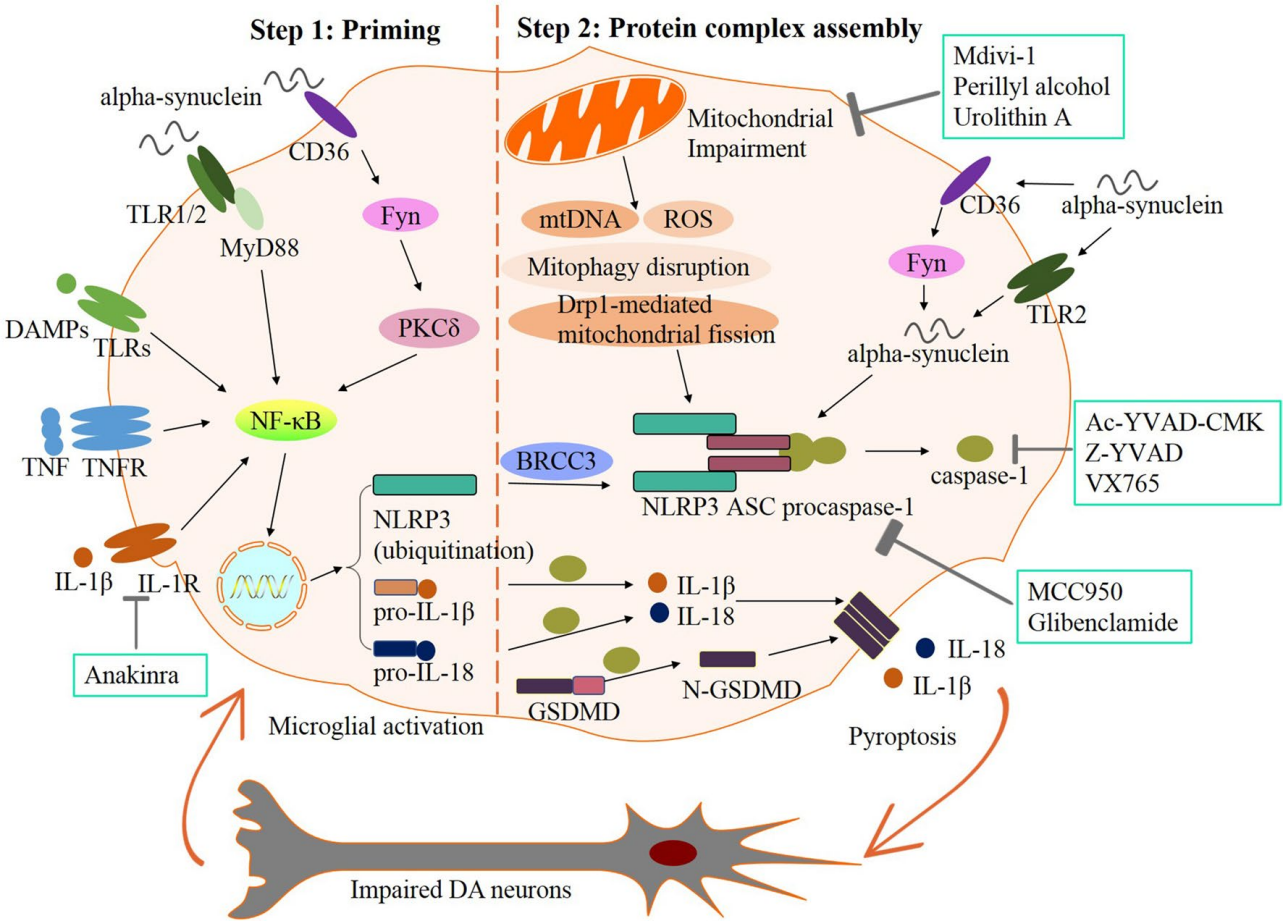
The mechanism underlying the mitochondrial impairment and NLRP3 inflammasome-mediated neuroinflammation in PD and the therapeutic approaches (green frames). In the priming process, alpha-synuclein is regulated by Fyn kinase in conjunction with CD36 to promote PKCδ-mediated NF-kB activation. Besides, alpha-synuclein can also interact with TLR1/2, leading to the nuclear translocation of NF-kB in a MyD88-dependent manner. In addition, TNFR, IL-1R, and other TLRs on microglia recognize TNF, IL-18, or DAMPs released from damaged neurons, inducing activation of the NF-kB pathway, subsequently leading to upregulated expression of NLRP3, pro-IL-1β, and pro-IL-18. In the protein complex assembly process, the alpha-synuclein is taken up by microglial cells in the CD36 or TLR2-mediated pathway. The mitochondrial impairment by excessive ROS and mtDNA, disruption of mitophagy, and Drp1-mediated mitochondrial fission promote activation of the NLRP3 inflammasome. ROS and alpha-synuclein fibrils stimulate the deubiquitination of NLRP3 through BRCC3 deubiquitinase, resulting in the formation of the NLRP3-ASC-procaspase-1 complex. After that, the inflammasome complex activates caspase-1, consequently prompting the production of mature IL-1β and IL-18 and the formation of plasma membrane pores that mediate pyroptosis and result in the secretion of IL-1β and IL-18, which exert inflammatory effects that damage DA neurons. Impaired DA neurons directly lead to microglial activation, producing large amounts of pro-inflammatory cytokines, then conversely contributing to the apoptosis of DA neurons. NLRP3, nucleotide-binding oligomerization domain-, leucine-rich repeat-, and pyrin domain-containing protein 3; PD, Parkinson's disease; PKCδ, protein kinase C-delta; NF-kB, nuclear factor-kappa B; TLR, Toll-like receptor; TNFR, tumor necrosis factor receptor; IL-1R, Interleukin-1 receptor; TNF, tumor necrosis factor; IL-18, interleukin-18; DAMPs, danger-associated molecular patterns; IL-1β, interleukin-1beta; ROS, reactive oxygen species; Drp1, dynamin-related protein 1; BRCC3, BRCA1-BRCA2-containing complex subunit 3; ASC, apoptosis-associated speck-like protein containing a caspase recruitment domain; DA, dopamine; GSDMD, gasdermin D.

An animal model study revealed that the NLRP3 inflammasome, not the NLRP1, NLRP2, NLRC4, and AIM2 inflammasomes, might be the pivotal complex that boosts 1-methyl-4-phenyl-1,2,3,6-tetrahydropyridine (MPTP)-induced pathogenesis. MCC950, an NLRP3 inhibitor, reverses MPTP-induced nigrostriatal damage [55]. There is evidence that abnormal aggregation of alpha-synuclein protein activates microglial cells and stimulates the NLRP3 pathway [23]. Activation of the NLRP3 inflammasome prompts the maturation of caspase-1, which causes the release of pro-inflammatory cytokines such as IL-1β and IL-18, thus inducing pyroptosis [56]. The expression level of oligomerized and phosphorylated alpha-synuclein, as well as IL-1β, in the peripheral blood of PD patients, is remarkably elevated [57]. Besides, researchers have reported upregulated gene expression of NLRP3, ASC, and caspase-1 and elevated protein expression of NLRP3, caspase-1, and IL-1β in peripheral blood mononuclear cells from PD patients [58]. Similarly, another study demonstrated that PD patients have significantly higher cerebrospinal fluid (CSF) concentrations of IL-1β and IL-18 than healthy controls [59]. Thus, NLRP3 inflammasome-mediated neuroinflammation plays an important role in PD progression. As the activation of the NLRP3 inflammasome requires a priming process (Step 1) and a protein complex assembly process (Step 2), we present the mechanisms underlying NLRP3 inflammasome activation in PD during these two steps, as shown in Fig. 1.

### Priming Process in PD

The pathological hallmark of PD is Lewy bodies, constituted by fibrillar alpha-synuclein [60]. Under normal circumstances, alpha-synuclein is expressed in synaptic terminals and participates in synaptic function [61]. There is evidence that alpha-synuclein is strikingly expressed in the neurons of the brain regions affected by PD, such as the substantia nigra pars compacta, dorsal motor nucleus of the vagus, and olfactory bulb [62]. The levels of both oligomers and phosphorylated alpha-synuclein are significantly elevated in the peripheral plasma of PD patients [57]. Extracellular alpha-synuclein that is released from neuronal cells can activate the inflammatory responses in microglia, contributing to PD progression [63]. The use of alpha-synuclein in the development of animal models of PD has become popular in recent years, including the injection of alpha-synuclein pre-formed fibrils model and the recombinant adeno-associated virus vector-mediated alpha-synuclein overexpression model [64]. In the clinic, alpha-synuclein may be helpful as a biomarker for PD, given its role in pathogenesis [65]. Total alpha-synuclein, as well as its phosphorylated and oligomeric forms in CSF, plasma, and saliva, can be detected by ELISA, mass spectrometry, or western blot [66, 67]. Extracellular alpha-synuclein may act as a DAMP for microglia. Alpha-synuclein can be regulated by Fyn kinase, a non-receptor Src family tyrosine kinase, in conjunction with the class B scavenger receptor CD36, to promote protein kinase C-delta-mediated NF-kB activation, which contributes to NLRP3 inflammasome priming [68]. Alpha-synuclein can also interact with the heterodimer Toll-like receptor 1 and 2 (TLR1/2) at the cell membrane, leading to the nuclear translocation of NF-kB in a MyD88-dependent manner [69]. In addition, the TLR2 or other TLRs on microglia can recognize the extracellular DAMPs released from damaged neurons, inducing activation of the NF-kB pathway [51], thus resulting in persistent neuroinflammation. Although the DAMPs may not be initiating factors in PD, they appear to be co-factors in its progression [70]. In addition, the receptors TNFR and IL-1 receptor (IL-1R) induce the priming process once stimulated by their ligands TNF or IL-1β, leading to activation of the NF-kB pathway [51]. NF-kB appears to play an important role in activating and regulating neuroinflammation in PD [71]. Activation of the NF-kB pathway subsequently leads to upregulated expression of NLRP3, pro-IL-1β, and pro-IL-18 [28].

### Protein Complex Assembly Process in PD

The priming process leads to the production of NLRP3, pro-IL-1β, and pro-IL-18. However, as ubiquitination of NLRP3 prevents its oligomerization with ASC, it is preactivated in this state. The ROS and neurotoxic alpha-synuclein fibrils called the second signal of the protein complex assembly process, stimulate the deubiquitination of NLRP3 through BRCA1-BRCA2-containing complex subunit 3 (BRCC3) deubiquitinase, as well as activate the nucleation of the inflammasome with ASC, resulting in the formation of the NLRP3–ASC–procaspase-1 complex [51]. A study found that BRCC3 expression is increased in PD models, whereas knocking it down with shRNA lentivirus decreases the NLRP3 inflammasome activity. Thus, BRCC3 may contribute to regulating the NLRP3 inflammasome in PD [72]. Alpha-synuclein can be taken up by microglial cells in a TLR2-mediated endocytosis-dependent manner [63] or *via* the CD36 and Fyn kinase-mediated pathway [68]. Afterward, aggregated alpha-synuclein can act as an endogenous danger signal to induce NLRP3 inflammasome activation. The alpha-synuclein in microglia induces the propagation of mitochondrial ROS and consequently leads to the activation of the NLRP3 inflammasome [68]. In addition to ROS production, the phagocytosis of alpha-synuclein also promotes the release of cathepsin B into the cytosol, leading to NLRP3 inflammasome activation [73]. Then, the inflammasome complex results in the activation of caspase-1, consequently prompting the production of mature forms of IL-1β and IL-18 [38, 39]. The plasma membrane pores formed by the oligomerization of N-GSDMD mediate pyroptosis and result in the secretion of mature IL-1β and IL-18, which exert inflammatory effects that damage DA neurons. It has been reported that primary human microglia exposed to alpha-synuclein fibrils significantly induce inflammasome assembly and secretion of IL-1β, demonstrating the similar mechanisms in primary human and mouse microglia in the activation of the NLRP3 inflammasome by alpha-synuclein [74]. Microglial NLRP3 inflammasome activation plays an essential role in the dopaminergic neurodegeneration process [75].

However, studies have shown that DA neurons can inhibit NLRP3 inflammasome activation *via* the dopamine D1 receptor (DRD1) [76–78]. The administration of A68930, a DRD1-specific agonist, decreases the expression of NLRP3, caspase 1, and IL-1β and inhibits microglial activation [79]. Moreover, studies have found that DRD1 signaling inhibits NLRP3 inflammasome activation through cyclic adenosine monophosphate, which binds to NLRP3, leading to the ubiquitination and degradation of NLRP3 [80]. In addition to DRD1, the D2 receptor (DRD2) may also be involved in the DA-induced inhibition of the NLRP3 inflammasome [81]. The study indicates that DA may negatively regulate the K^+^ efflux-induced activation of the NLRP3 inflammasome, which contributes to the DA neuron degeneration in PD [81]. Thus, there is a mutual regulation between DA neurons and the NLRP3 inflammasome. Not only is the NLRP3 inflammasome able to damage DA neurons, but DA neurons can inhibit NLRP3 inflammasome activation. Therefore, microglial NLRP3 inflammasome-mediated neuroinflammation is a crucial element in the pathogenesis of PD.

## Mitochondrial Impairment in the NLRP3 Inflammasome-mediated Neuroinflammation in PD

Mitochondria comprise various proteins, a small number of which are encoded by the mitochondrial genome, called mtDNA, located in the matrix [82]. New mitochondria are produced through mitochondrial binary fission [83, 84]. The clearance of damaged or unwanted mitochondria, called mitophagy, is also required to maintain mitochondrial and cellular homeostasis [85]. The essential function of mitochondria is to produce energy [86]. They also produce many biosynthetic intermediates to mediate autophagy and apoptosis, store Ca^2+^ for cell signaling activities, and regulate cell growth. Thus, mitochondrial impairment plays a role in many diseases, including neurodegenerative diseases [87]. Studies are increasingly showing that the impairment of mitochondrial function augments NLRP3 inflammasome activation, triggering systemic or local inflammation [17, 18]. Autophagy negatively regulates NLRP3 inflammasome activity, while ROS positively regulates it [17]. After sensing mitochondrial impairment, the NLRP3 inflammasome promotes an inflammatory process, consequently augmenting mitochondrial damage [17]. There is evidence indicating that mitochondrial dysfunction aggravates the pro-inflammatory cascade mediated by the microglial NLRP3 inflammasome, contributing to the neurodegenerative process in DA neurons [88]. Thus, mitochondrial impairment in NLRP3 inflammasome-mediated neuroinflammation is of importance in PD.

Evidence indicates that mitochondria participate in immune system activation, inflammation, and the pathogenesis of inflammatory diseases through endogenous mitochondrial damage-associated molecular patterns (mtDAMPs) that include ATP, mtDNA, mitochondrial ROS, mitochondrial N-formyl peptides, and mitochondrial cardiolipin, which are released to recognize PRRs during mitochondrial damage [89]. Accumulating studies in PD patients and animal PD models indicate that mitochondrial impairment participates in PD progression. In both sporadic and familial forms of PD, mitochondrial dysfunction includes electron transport chain impairment, mitochondrial morphology, alterations in dynamics, disruption of mitochondrial homeostasis and biogenesis, deficiency of mitochondrial oxidative phosphorylation, defective mitophagy, mtDNA mutations, and Ca^2+^ imbalance, resulting in a decline in energy generation and ROS production, as well as increased apoptosis [90–93]. The mitochondrial impairment, oxidative stress, and protein turnover defect induce DA neuron death [16]. A study indicates that MPTP causes NLRP3 inflammasome activation in PD [94]. MPTP is a neurotoxin that induces mitochondria damage, which might activate the NLRP3 inflammasome by mitochondrial DAMPs, such as excessive ROS and mtDNA. In addition, a study revealed that rotenone augments dynamin-related protein 1 (Drp1)-mediated mitochondrial fission, which induces mitochondrial dysfunction. Moreover, Drp1-mediated mitochondrial fission promotes the nuclear translocation of NF-kB and activation of the NLRP3 inflammasome [95]. Similarly, another study demonstrated that rotenone aggravates NLRP3 inflammasome activation through mitochondrial dysfunction. The researchers found that mitochondrial dysfunction in microglia triggers NLRP3 inflammasome activation, aggravating the process of DA neuron degeneration [88]. Activation of the NLRP3 inflammasome may require a signal from mitochondria damage. Furthermore, pharmacological attenuation of mitochondria damage inhibits microglial activation and the NLRP3 inflammasome pathway, promoting neuronal survival in PD [96]. In addition, mitophagy is involved in the quality control of mitochondria. Disruption of mitophagy can lead to NLRP3 inflammasome activation in microglia and neurodegeneration in PD [97]. Thus, mitochondrial impairment is critically involved in NLRP3 inflammasome-mediated neuroinflammation in PD.

The role of the alpha-synuclein/TLRs/NF-kB/NLRP3 inflammasome axis and microglial activation in PD has been elucidated in a recent review [98]. The review revealed that alpha-synuclein interacts with TLR1/2, TLR2, and TLR4, triggering NF-kB-dependent upregulation of NLRP3, activation of the NLRP3 inflammasome, and the production of pro-inflammatory cytokines [98]. In this present review, regarding the initiation of the priming process, we do not only focus on the TLR signaling pathway but also CD36, as well as the TNFR and IL-1R signaling pathways. Taken together, as shown in Fig. 1, in the priming process, the CD36, TLR1/2, TNFR, IL-1R, and other TLRs on microglia can recognize the alpha-synuclein, TNF, IL-18, or other DAMPs released from damaged neurons, inducing activation of the NF-kB pathway, subsequently leading to upregulated expression of NLRP3, pro-IL-1β, and pro-IL-18. In the protein complex assembly process, the alpha-synuclein is taken up by microglial cells in the CD36 or TLR2-mediated pathway, activating the nucleation of the inflammasome. Mitochondrial impairment, including excessive ROS and mtDNA, disruption of mitophagy, and Drp1-mediated mitochondrial fission, promotes activation of the NLRP3 inflammasome. After that, the inflammasome complex activates caspase-1, consequently prompting the cleavage of the pro-inflammatory cytokines IL-1β and IL-18 and the formation of plasma membrane pores that mediate pyroptosis and result in the secretion of IL-1β and IL-18, which exert inflammatory effects to damage DA neurons. Impaired DA neurons directly lead to microglial activation, producing large amounts of pro-inflammatory cytokines, then contributing to the apoptosis of DA neurons.

## The NLRP3 Inflammasome: A Target for Novel Therapeutic Approaches in PD

Currently, drugs that treat PD clinically are divided into three categories: direct supplementation of exogenous DA precursors such as levodopa, DA receptor agonists such as pramipexole, and monoamine oxidase B inhibitors such as selegiline. Among these, levodopa is the best option [99–101]. However, although these treatments are initially effective, they present many problems, such as low tolerance and long-term effects. Currently, no therapy can efficiently curb the progression of PD [2]. Informed by new insights into the involvement of mitochondrial impairment and NLRP3 inflammasome-mediated neuroinflammation in this disease, the therapeutic strategies targeting the NLRP3 inflammasome for PD treatment are discussed next, including the NLRP3 inflammatory pathway inhibitors, mitochondria-focused treatments, microRNA, and other therapeutic compounds.

### Inhibitors Targeting the NLRP3 Inflammasome

**Fig. 2:**
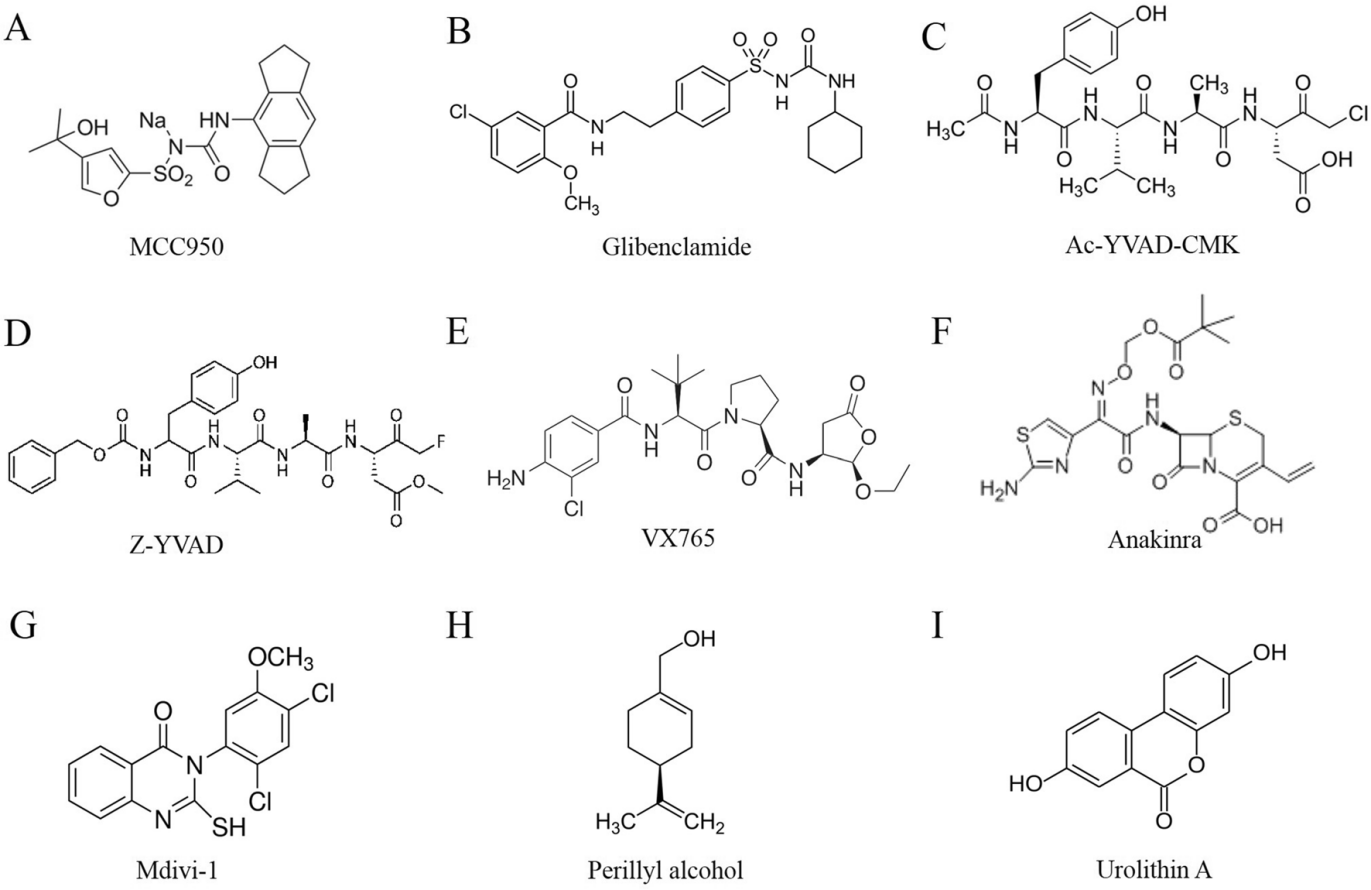
The structures of inhibitors of the NLRP3 inflammatory pathway (**A**–**F**) and mitochondria-focused therapeutic compounds **G**–**I** that target the NLRP3 inflammasome for the treatment of PD. **A** MCC950, **B** Glibenclamide, **C** Ac-YVAD-CMK, **D** Z-YVAD, **E** VX765, **F** Anakinra, **G** Mdivi-1, **H** Perillyl alcohol, **I** Urolithin A.

As the NLRP3 inflammasome plays a vital role in PD progression, inhibitors of NLRP3 inflammatory pathways that may hamper NLRP3 inflammasome activation offer novel therapeutic avenues for PD therapy. A study revealed that MCC950, an NLRP3 inhibitor, inhibits NLRP3 inflammasome activation and alleviates behavioral dysfunctions and DA neuronal degeneration in MPTP-administered mice [55]. Glibenclamide, another inhibitor of the NLRP3 inflammasome, blocks NLRP3 inflammasome activation, as demonstrated by decreased expression of NLRP3, activated caspase-1, and mature IL-1β in paraquat- and maneb-administered mice. Moreover, glibenclamide relieves the paraquat and maneb-induced microglial M1 pro-inflammatory reaction and NF-kB activation to protect DA neurons in PD mice [102]. In addition, Ac-YVAD-CMK, a caspase-1 inhibitor, suppresses the downstream pathway of the NLRP3–caspase-1–IL-1β axis in LPS- and 6-hydroxydopamine-induced PD rats, providing a new basis for PD therapy [103]. ZYVAD, another caspase-1 inhibitor, inhibits the caspase-7/poly (ADP-ribose) polymerase 1/apoptosis-inducing factor-mediated DA neuronal apoptosis pathway in PD mice [104]. Similarly, VX765, a potent caspase-1 inhibitor, decreases the truncation of alpha-synuclein into its highly aggregation-prone form, playing a neuroprotective role in a neuronal cell model of PD [105]. In addition, anakinra, an IL-1 receptor antagonist, inhibits the binding of IL-1β and its receptor to relieve the inflammatory effect of IL-1β and curb the progression of PD [106]. Thus, these inhibitors and antagonists are potential therapeutic drugs targeting the NLRP3 inflammasome in the treatment of PD. The structures of these inhibitors are shown in Fig. 2A–F.

### Mitochondria-Focused Treatments to Attenuate NLRP3 Inflammasome-mediated Neurodegeneration

It is known that mitochondrial dysfunction is critically involved in NLRP3 inflammasome-mediated neuroinflammation in PD, so it is a promising target for PD treatment [107]. As noted above, a study has revealed that rotenone augments Drp1-mediated mitochondrial fission, and this, in turn, induces mitochondrial dysfunction, promoting the nuclear translocation of NF-kB and activation of the NLRP3 inflammasome [95]. Mdivi-1, a selective Drp1 inhibitor, significantly attenuates the morphological disruption of mitochondria and Drp1 translocation, negatively regulates the nuclear translocation of NF-kB, and eventually curbs activation of the NLRP3 inflammasome [95]. NLRP3 inflammasome activation may require a signal from mitochondria damage. Moreover, pharmacological treatment, such as perillyl alcohol, inhibits the nuclear translocation of NF-kB, promotes the translocation of PARKIN into mitochondria, and maintains cellular redox homeostasis, inhibiting microglial activation and the NLRP3 inflammasome pathway and promoting neuronal survival in PD [96]. Mitophagy contributes to mitochondrial quality control, and its disruption leads to activation of the microglial NLRP3 inflammasome and neurodegeneration in PD. Urolithin A, a natural compound produced by gut bacteria, increases mitophagy. A study has shown that urolithin A restores mitochondrial function and inhibits NLRP3 inflammasome activation, protecting against dopaminergic neurodegeneration in PD [97]. Thus, these potential mitochondria-focused treatments may be promising strategies to influence the activation of the NLRP3 inflammasome in PD. The structures of the mitochondria-focused therapeutic compounds that target the NLRP3 inflammasome for PD treatment are shown in Fig. 2G–I.

### MicroRNA and Other Therapeutic Compounds Targeting the NLRP3 Inflammasome

Recently, accumulating evidence has indicated that microRNA is involved in inflammatory responses. A study has shown that miR-188-3p-enriched exosome treatment inhibits autophagy and pyroptosis by targeting NLRP3 and CDK5 in MPTP-treated mice and MN9D cells [108]. Besides, a study found that miR-124-3p is the target of the long non-coding RNA (lncRNA) HOXA11-AS and follistatin-like protein 1 (FSTL1). Inhibiting HOXA11-AS suppresses neuroinflammation and neuronal apoptosis *via* the miR-124-3p–FSTL1–NF-kB–NLRP3 inflammasome axis in PD mice [109]. In addition, researchers have found that miR-135b mimics suppresses MPP(+)-induced pyroptosis and the upregulation of thioredoxin-interacting protein (TXNIP), NLRP3, caspase-1, ASC, N-GSDMD, and IL-1β by targeting forkhead box 1 [110]. Moreover, miR-190 alleviates neuronal damage and inhibits inflammation by inhibiting the expression and activation of the NLRP3 inflammasome in MPTP-induced PD mice [111]. In addition, the miR-30e agomir significantly suppresses the expression and activation of the NLRP3 inflammasome, attenuates the release of inflammatory cytokines, inhibits DA neuron loss, and improves behavioral deficits in MPTP-induced PD mice [112]. Similarly, miR-29c-3p negatively regulates NLRP3 inflammasome activation by targeting the nuclear factor of activated T cells 5 (NFAT5) to exert anti-inflammatory effects in PD animals and neuronal models [113]. In addition, microRNA-7 mimics inhibit the neuroinflammation mediated by microglial NLRP3 inflammasome activation in MPTP-induced PD mice [114]. Lastly, targeting miR-326 [115] or miR-1301-3p [116] may inhibit NLRP3 inflammasome activation in PD. Thus, these microRNAs are potential targets for PD therapy.

In addition, some studies have revealed that other exogenous compounds indirectly attenuate NLRP3 inflammasome-mediated neuroinflammation in PD. For example, GW501516, a peroxisome proliferator-activated receptor β/δ agonist, inhibits NLRP3-mediated neuroinflammation in MPTP model mice [117]. Besides, melatonin remarkably suppresses microglial activation and NLRP3 inflammasome activity, reduces DA neuron damage, and alleviates behavioral dysfunction *via* a silence information regulator 1-dependent pathway in MPTP-induced PD models [118]. Moreover, N-methyl-4-isoleucine-cyclosporine (NIM811), a derivative of the immunosuppressant cyclosporin A, passes through the blood-brain barrier (BBB). The researchers found that NIM811 inhibits rotenone-induced NLRP3 inflammasome activation and pyroptosis [119]. Therefore, these compounds are potential therapeutic drugs in PD treatment. In addition, it should be noted that a recent review demonstrated that microglial autophagy plays a role in maintaining brain homeostasis and negatively regulates NLRP3 inflammasome-mediated neuroinflammation [120]. Autophagy, a lysosomal degradation pathway, keeps cells functioning normally by removing misfolded or aggregated proteins, such as alpha-synuclein, and clearing damaged organelles, such as mitochondria, endoplasmic reticulum, and peroxisomes, as well as eliminating intracellular pathogens [121, 122]. Studies have shown that disruption of autophagy is a contributor to age-related pathologies and cognitive and motor dysfunction [123]. By stimulating microglial autophagy, autophagy inducers eliminate the misfolded proteins, remove damaged mitochondria, and degrade the NLRP3 inflammasome and its components. The previously noted review laid a foundation for using NLRP3 inflammasome inhibitors in combination with autophagy inducers. And this combination therapy may be more effective than a single treatment in treating neurodegenerative diseases [120]. Thus, combination therapy may inhibit the NLRP3 inflammasome-mediated neuroinflammation in a more efficient manner, which requires further research.

In this review, the therapeutic strategies targeting NLRP3 inflammasome for the treatment of PD are discussed, including the inhibitors of NLRP3 inflammatory pathways, mitochondria-focused treatments, microRNAs, and other therapeutic molecules. The pharmacological activities of the therapeutic compounds that target the NLRP3 inflammasome for PD treatment are summarized in Table 1.

**Table 1:** The pharmacological activities of the therapeutic compounds that target the NLRP3 inflammasome for PD treatment.

Classification	Drugs	Cell lines/ Animal models	Administration Dose/Time/Route	Mechanisms of action	References
Targeting NLRP3 inflammasome	MCC950	MPTP-induced PD mice	10 mg/kg, daily for 13 days, i.p.	Inhibits the activation of glial cells and the NLRP3 inflammasome	[55]
	Glibenclamide	Paraquat and maneb-induced PD mice	1 mg/kg, 8 weeks (twice per week), i.p.	Blocks NLRP3 inflammasome activation, relieves microglial M1 pro-inflammatory reaction, and NF-kB activation	[102]
	Ac-YVAD-CMK	LPS- and 6-OHDA-induced PD rats	300 or 1200 ng/rat, daily for 6 days (twice on day 1), injection at left SNc	A caspase-1 inhibitor suppresses the downstream pathway of the NLRP3 inflammasome	[103]
	Z-YVAD	MPP^+^-treated SH-SY5Y cells	Cells were pre-incubated with 10 μmol/L Z-YVAD for 1 h	A caspase-1 inhibitor, inhibits the caspase-7/PARP1/AIF-mediated DA neuronal apoptosis pathway	[104]
	VX765	Parental BE (2)-M17 human dopaminergic neuroblastoma cells overexpressing alpha-synuclein	M17- alpha-synuclein cells were treated with VX765	A potent caspase-1 inhibitor decreases the truncation of alpha-synuclein into its highly aggregation-prone form	[105]
	Anakinra	Caco-2 cells were cultured with alpha-synuclein plus LPS-conditioned medium.	Caco-2 cells were treated with 100 ng/mL anakinra for 72 h	An IL-1Ra inhibits the binding of IL-1β and its receptor	[106]
Mitochondria-focused treatments	Mdivi-1	Rotenone-induced PD rats	20 mg/kg, once daily, injection beginning on the day of rotenone treatment (3 weeks) and continued until the last behavior test, i.p.	A selective Drp1 inhibitor attenuates the mitochondria morphological disruptions and Drp1 translocation, negatively regulates the NF-kB nuclear translocation, and eventually curbs the NLRP3 inflammasome activation	[95]
	Perillyl alcohol	MPTP-induced PD mice, LPS and H_2_O_2_-treated N9 mouse microglial cells	Mice: 100 or 200 mg/kg, daily for 14 days, orally	Inhibits the nuclear translocation of NF-kB, promotes PARKIN translocation into mitochondria, maintains cellular redox homeostasis, then inhibits microglial activation and the NLRP3 inflammasome pathway and promotes neuronal survival	[96]
			Cells: 100 or 200 μmol/L		
	Urolithin A	MPTP-induced PD mice, LPS-treated BV2 microglial cells	Mice: 20 mg/kg, daily for 7 days, i.p.	Restores mitochondrial function and inhibits NLRP3 inflammasome activation, protecting against dopaminergic neurodegeneration	[97]
			Cells: 2.5, 5, and 10 μmol/L for 2 h		
Other compounds	GW501516	MPTP-induced PD mice	60, 120, or 240 μg per mouse, once, 24 h before MPTP administration, ICV injection into the left lateral ventricle	A PPARβ/δ agonist, inhibits NLRP3-mediated neuroinflammation	[117]
	Melatonin	MPTP-induced PD mice, MPP^+^-treated BV2, and primary microglia cells	Mice: 10 mg/kg, daily for 5 days, 1 h before each MPTP injection, i.p.	Suppresses microglial activation and NLRP3 inflammasome activity, and reduces dopaminergic neuron damage via a SIRT1-dependent pathway	[118]
			Cells: 50, 100, or 400 μmol/L for 0–6 h.		
	NIM811	Rotenone-treated murine hippocampal HT22 cells	50, 100, 200, 400, or 800 n mol/L for 3 h	A derivative of the immunosuppressant CsA inhibits NLRP3 inflammasome activation and pyroptosis	[119]

i.p., intraperitoneal injection; ICV, intracerebroventricular injection; NLRP3, nucleotide-binding oligomerization domain-, leucine-rich repeat-, and pyrin domain-containing protein 3; PD, Parkinson's disease; MPTP, 1-methyl-4-phenyl-1,2,3,6-tetrahydropyridine; NF-kB, nuclear factor-kappa B; 6-OHDA, 6-hydroxydopamine; SNc, substantia nigra pars compacta; PARP1, poly (ADP-ribose) polymerase 1; AIF, apoptosis-inducing factor; DA, dopamine; LPS, lipopolysaccharides; IL-1Ra, IL-1 receptor antagonist; IL-1β, interleukin-1beta; Drp1, dynamin-related protein 1; PPARβ/δ, peroxisome proliferator-activated receptor β/δ; SIRT1, silence information regulator 1; CsA, cyclosporin A

## Research Progress in Clinical Trials Targeting the NLRP3 Inflammasome

In 2015, a study published in *Nature Medicine* revealed that MCC950, a small-molecule inhibitor of the NLRP3 inflammasome, can be used to treat inflammatory diseases [124]. Afterward, the research on NLRP3 inhibitors entered the clinical stage. Inflazome, IFM Tre, and Olatec are the three representative companies. Inflazome, acquired by Roche, generated three kinds of the compound with distinctive tissue distribution properties based on MCC950 [125]: inzomelid, a central nervous system (CNS) penetrant NLRP3 inhibitor, is being investigated for Alzheimer's disease, PD, cryopyrin-associated periodic syndrome, and amyotrophic lateral sclerosis; Somalix, a peripherally restricted NLRP3 inhibitor, is for cryopyrin-associated periodic syndrome; in addition, gut-restricted compounds are in development. IFM Tre, acquired by Novartis, developed IFM-2747, which is somewhat differentiated from MCC950, to treat gout, coronary artery disease, non-alcoholic fatty liver disease, and Crohn’s disease [125]. Olatec developed dapansutrile, a simple β-sulfonyl nitrile and the most advanced NLRP3 inhibitor in clinical trials, to treat acute gout [126]. NLRP3 seems to be a promising drug target; however, it faces some problems. As the NLRP3 inflammasome plays a vital role in defense against various infections, its inhibitors may disturb the host response. Evidence has shown that inhibition of NLRP3 disrupts the host response to influenza A infection [127], *Streptococcus pneumoniae* infection [128], and fungal infection [129]. Besides, since PD is a degenerative disorder of the CNS, it is necessary to improve the BBB penetration of NLRP3 inhibitors. Small molecules can be structurally modified to increase their BBB penetration by improving diffusion (increasing lipophilicity, reducing hydrogen bond donor capacity, reducing topological polar surface area, enhancing rigidity, reducing p*K*_a_, and controlling multiple parameters), reducing efflux (multiple drug resistance 1 and breast cancer resistance protein), and activating carrier transporters (L-type amino-acid transporter and glucose transporter 1) [130]. Future small molecule CNS drug development can refer to these strategies. Taken together, NLRP3 possesses a complex activation mechanism, thus NLRP3 inhibitors need to be further explored.

## Conclusion

Alpha-synuclein participates in NLRP3 inflammasome-mediated neuroinflammation in microglia and is a crucial element in the pathogenesis of PD. It has been shown that several risk factors in PD modulate immune function, such as *Leucine-rich repeat kinase 2 (LRRK2), Synuclein Alpha (SNCA), glucocerebrosidase (GBA), Parkin RBR E3 Ubiquitin Protein Ligase (PRKN),* and *PTEN-induced kinase 1 (PINK1)* [8, 131]. These risk factors contribute to PD pathogenesis. For example, *LRRK2* and *SNCA* are involved in vesicular trafficking, *LRRK2* and *GBA* are involved in autophagy, *LRRK2* is involved in mitochondrial function, and *GBA* is involved in the lysosomal process [132]. Our previous review reported that, as a non-motor manifestation of PD, intestinal dysfunction might affect the intestinal microenvironment to influence the CNS through alpha-synuclein pathology and systemic inflammation [133]. Specifically, the *NLRP3* gene may be one of the mechanisms linking intestinal inflammation and PD [133]. It should be noted that NLRP3 inflammasome-mediated neuroinflammation in microglia occurs in the early stages of PD [134]. Positron emission tomography (PET) examination has also revealed that microglial activation appears at very early or preclinical stages of PD [7]. In addition to microglia, NLRP3 inflammasome-mediated neuroinflammation may also occur in mesencephalic neurons. A study demonstrated that, in PD patients, the expression of NLRP3 is elevated in mesencephalic neurons [135]. Besides, it has been shown that *NLRP3* rs7525979, a synonymous single-nucleotide polymorphism, significantly reduces PD risk by altering the translation efficiency of NLRP3 [135]. Furthermore, evidence indicates that mitochondrial dysfunction aggravates the microglial NLRP3 inflammasome-mediated pro-inflammatory cascade, contributing to the DA neuron neurodegenerative process in PD [88]. In summary, the microglial NLRP3 inflammasome and the related mitochondrial impairment are crucial elements in the pathogenesis of PD and might be promising targets for PD therapy. The therapeutic strategies targeting the NLRP3 inflammasome for PD treatment include inhibitors of the NLRP3 inflammatory pathways, mitochondria-focused treatments, microRNAs, and other therapeutic compounds. However, even although inhibiting NLRP3 inflammasome activation has been shown to have therapeutic effects in PD animal models, the safety and effectiveness of these potential drugs need to be confirmed in PD patients in clinical trials. We should accelerate the progress of transforming basic research into clinical applications to achieve novel therapeutic strategies for PD.
